# Blood protein assessment of leading incident diseases and mortality in the UK Biobank

**DOI:** 10.1038/s43587-024-00655-7

**Published:** 2024-07-10

**Authors:** Danni A. Gadd, Robert F. Hillary, Zhana Kuncheva, Tasos Mangelis, Yipeng Cheng, Manju Dissanayake, Romi Admanit, Jake Gagnon, Tinchi Lin, Kyle L. Ferber, Heiko Runz, Biogen Biobank Team, Kyle L. Ferber, Christopher N. Foley, Riccardo E. Marioni, Benjamin B. Sun

**Affiliations:** 1 Optima Partners, Edinburgh, UK; 2 Centre for Genomic and Experimental Medicine, Institute of Genetics and Cancer, University of Edinburgh, Edinburgh, UK; 3 Bayes Centre, University of Edinburgh, Edinburgh, UK; 4 Biostatistics, Research and Development, Biogen Inc., Cambridge, MA USA; 5 Translational Sciences, Research and Development, Biogen Inc., Cambridge, MA USA; 6 Cardiovascular Epidemiology Unit, Department of Public Health and Primary Care, University of Cambridge, Cambridge, UK

**Keywords:** Predictive markers, Diseases, Ageing

## Abstract

The circulating proteome offers insights into the biological pathways that underlie disease. Here, we test relationships between 1,468 Olink protein levels and the incidence of 23 age-related diseases and mortality in the UK Biobank (*n* = 47,600). We report 3,209 associations between 963 protein levels and 21 incident outcomes. Next, protein-based scores (ProteinScores) are developed using penalized Cox regression. When applied to test sets, six ProteinScores improve the area under the curve estimates for the 10-year onset of incident outcomes beyond age, sex and a comprehensive set of 24 lifestyle factors, clinically relevant biomarkers and physical measures. Furthermore, the ProteinScore for type 2 diabetes outperforms a polygenic risk score and HbA1c—a clinical marker used to monitor and diagnose type 2 diabetes. The performance of scores using metabolomic and proteomic features is also compared. These data characterize early proteomic contributions to major age-related diseases, demonstrating the value of the plasma proteome for risk stratification.

## Abstract (web summary)

Gadd et al. identify proteins circulating in the blood that can stratify the risk people have of developing a range of leading age-related diseases, up to a decade before onset.

## Main

Identifying individuals who are at a high risk of age-related morbidities may aid in personalized medicine. Circulating proteins can discriminate disease cases from controls and delineate the risk of incident diagnoses^1–8^. While singular protein markers offer insight into the mediators of disease^5,9–11^, simultaneously harnessing multiple proteins may improve clinical utility^12^. Clinically available non-omics scores such as QRISK typically profile the 10-year onset risk of a disease^13^. Proteomic scores have recently been trained on diabetes, cardiovascular and lifestyle traits as outcomes in 16,894 individuals^14^. Proteomic and metabolomic scores have also been developed for time-to-event outcomes, including all-cause mortality^6,15–21^.

**Extended Data Fig. 1:**
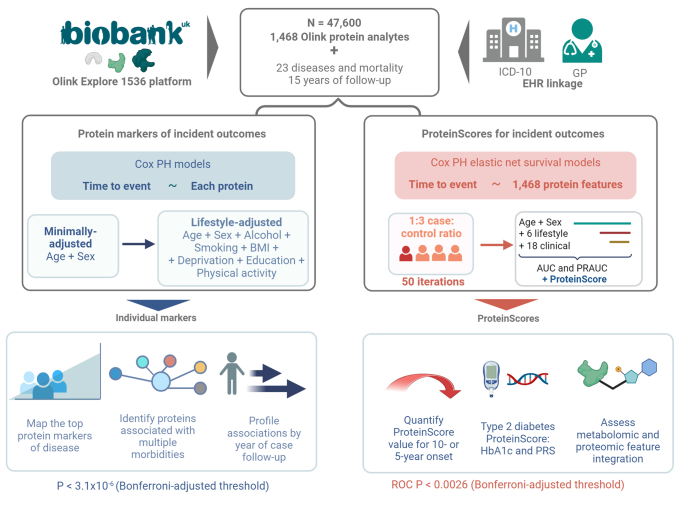
Study design summary for protein assessment of leading incident diseases in the UK Biobank (N=47,600). Individual Cox proportional hazards (PH) models were used to profile relationships between baseline protein analytes and incident diseases or death, over a maximum of 15 years of electronic health linkage pertaining to cases. Associations that had P < 3.1x10^−6^ (Bonferroni-adjusted threshold) in minimally-adjusted (age and sex) and lifestyle-adjusted models were retained. Proteins associated with multiple morbidities were identified and associations were explored by year of case follow-up. Next, proteomic predictors (ProteinScores) were trained using Cox PH elastic net regression for 19 of the incident outcomes with a minimum of 150 cases. All ProteinScores were developed for 10-year onset of disease, except endometriosis, cystitis and amyotrophic lateral sclerosis that had case distributions that were better-suited to 5-year assessment (80% of cases diagnosed by year 8 of follow-up). Of fifty ProteinScore iterations with randomly sampled train and test populations, the ProteinScore with median improvement in AUC beyond a minimally-adjusted model was selected. Improvements in AUC due to adding the ProteinScores into models with increasingly complex covariate structures were quantified. The type 2 diabetes trait was taken forward as a case study to explore the potential value ProteinScores may offer, in the context of HbA1c (a clinically used biomarker) and a polygenic risk score (PRS). Integration of metabolomics features for scoring was investigated for death and type 2 diabetes outcomes as case studies. Created with BioRender.com.

Here, we demonstrate how large-scale proteomic sampling can identify candidate protein targets and facilitate the prediction of leading age-related incident outcomes in mid to later life (see the study design summary in Extended Data Fig. 1). We used 1,468 Olink plasma protein measurements in 47,600 individuals (aged 40–70 years) available as part of the UK Biobank Pharma Proteomics Project (UKB-PPP)^22^. Cox proportional hazards (PH) models were used to characterize associations between each protein and 24 incident outcomes, ascertained through electronic health data linkage. Next, the dataset was randomly split into training and testing subsets to train proteomic scores (ProteinScores) and assess their utility for modeling either the 5- or 10-year onset of the 19 incident outcomes that had a minimum of 150 cases available. We modeled ProteinScores alongside clinical biomarkers, polygenic risk scores (PRS) and metabolomics measures to investigate how these markers may be used to augment risk stratification.

## Results

### The UKB-PPP sample

**Extended Data Fig. 2:**
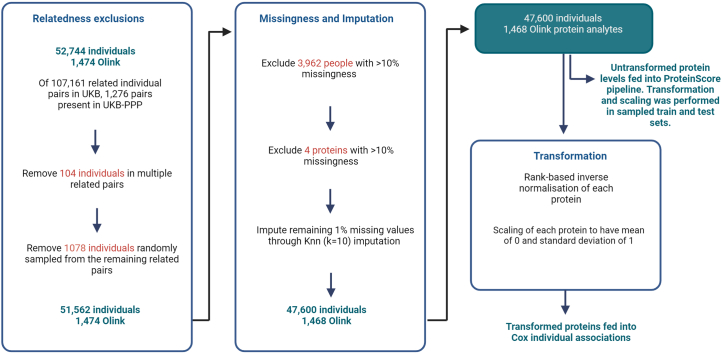
Summary of processing steps applied to the protein measurement data in UKB-PPP. Related individuals were excluded, leaving a dataset containing 51,562 individuals with 1,474 Olink protein analytes measured. Next, 3,962 individuals that had >10% missing data were excluded, followed by four proteins that had >10% missing data. The remaining missing protein measurements (1% of total measurements) were imputed through K-nearest neighbours (Knn; k=10) imputation. The final dataset was comprised of 47,600 individuals and 1,468 Olink protein analytes. Protein levels were rank-based inverse normalised and scaled to have a mean of 0 and standard deviation of 1 prior to individual Cox PH analyses. Untransformed protein levels were fed into the model pipeline for ProteinScore development and were rank-based inverse normalised and scaled to have a mean of 0 and standard deviation of 1 in train and test sets separately once these were sampled for each outcome. Created with BioRender.com.

In this study, data on 1,468 protein analytes (Supplementary Table 1) measured at baseline in 47,600 unrelated individuals ranging in age between 40 and 70 years (Supplementary Table 2) were used. Further details on the preparation pipeline are summarized in Extended Data Fig. 2 and the Supplementary Note. Principal component analyses indicated that the first 678 components explained a cumulative variance of 90% in the protein levels (Supplementary Table 3).

### Protein associations with incident outcomes

We identified differential plasma protein levels that were associated with the onset of 23 diseases (including leading causes of disability and reductions in healthy life expectancy)^23–25^ and all-cause mortality (Table 1). The maximal follow-up period was 15 years across the 24 outcomes.

**Table 1:** The 24 incident outcomes profiled over a maximum of 15 years of follow-up in the UK Biobank (*n* = 47,600)

Incident diagnosis	Incident cases (*n*)	Controls (*n*)	Mean years to incident case diagnosis (s.d.)
Schizophrenia	54	47,449	6.5 (3.4)
Brain/CNS cancer	82	47,507	5.5 (2.8)
Multiple sclerosis	96	47,165	5.6 (3.2)
Major depression	111	47,229	4.2 (3.1)
Systemic lupus erythematosus	134	47,096	5.1 (2.6)
Endometriosis^a^	157	24,768	4.8 (3.3)
Vascular dementia^b^	195	33,907	8.1 (3)
Gynecological cancer^a^	256	25,185	5 (3)
Amyotrophic lateral sclerosis	264	47,269	5.4 (2.7)
Inflammatory bowel disease	275	46,727	5.9 (3.3)
Lung cancer	403	47,158	5.9 (3.2)
Liver disease	432	47,104	7 (3.3)
Alzheimer’s dementia^b^	446	33,642	7.8 (2.8)
Colorectal cancer	508	46,890	5.8 (3.1)
Cystitis^a^	531	24,160	4.1 (3)
Rheumatoid arthritis	593	46,310	6.8 (3.2)
Parkinson’s disease	659	46,802	5.4 (3.2)
Ischemic stroke	765	46,657	6.8 (3.4)
Breast cancer^a^	772	24,086	5.2 (3.1)
Prostate cancer^a^	1,001	20,628	5.7 (3.1)
COPD	1,998	44,948	6.3 (3.4)
Type 2 diabetes	2,822	43,370	6 (3.3)
Ischemic heart disease	3,338	41,341	6.3 (3.4)
Death	4,445	43,155	7.9 (3.5)

Counts for incident cases and controls are provided, with mean years to diagnosis for incident cases. These data were used in individual Cox PH models to identify protein levels that were associated with incident outcomes. CNS, central nervous system.

^a^Sex-stratified traits.

^b^Alzheimer’s and vascular dementias were restricted to individuals aged 65 years or older at the time of diagnosis for cases or at the time of censoring for controls.

**Fig. 1:**
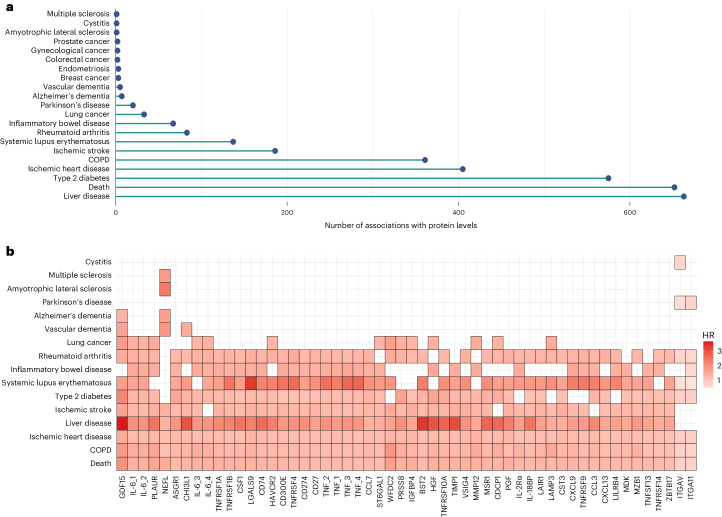
Individual protein associations with incident outcomes in the UK Biobank (*n* = 47,600). **a**, Number of associations between protein analytes and time to onset for 21 outcomes that had *P* < 3.1 × 10^−6^ (Bonferroni-adjusted threshold) in both basic and fully adjusted Cox PH models. There were 3,209 associations in total involving 963 protein analytes. Two-sided tests were used in all cases. **b**, HR per 1 s.d. higher level of the transformed protein analytes (compared within individuals at baseline). Fifty-four protein analytes that were associated with eight or more outcomes in the individual Cox PH models are shown. Each association is represented by a rectangle. Cox PH models were adjusted for age, sex and six lifestyle factors (BMI, alcohol consumption, social deprivation, educational attainment, smoking status and physical activity). Every association identified for these proteins had HR > 1 (red), and associations are shaded based on the HR effect size (darkest coloration indicating a larger magnitude of effect). The largest HR shown is for the association between GDF15 levels and liver disease (HR = 3.7). Source data

In minimally adjusted (age- or age- and sex-adjusted) models, there were 5,273 significant associations between 1,211 unique proteins and 23 outcomes (Bonferroni-adjusted *P* value threshold = 3.1 × 10^−6^) (Supplementary Table 4). Upon further adjustment for health and lifestyle risk factors (body mass index (BMI), alcohol consumption, social deprivation, education status, smoking status and physical activity), there were 3,209 associations with *P* < 3.1 × 10^−6^ (Fig. 1a and Supplementary Table 5).

These 3,209 associations involved 963 unique protein analytes and 21 outcomes, ranging from 1 association for amyotrophic lateral sclerosis, cystitis and multiple sclerosis to 652 and 663 associations for mortality and liver disease, respectively (Supplementary Table 6).

Fifty-four proteins had significant associations with eight or more incident morbidities (Fig. 1b); in all instances, higher levels of the proteins at baseline were associated with a higher risk of disease or death (that is, hazard ratio (HR) > 1). Of the 54 proteins, growth differentiation factor 15 (GDF15) had the largest number of associations (11 incident outcomes), followed by interleukin-6 (IL-6) and plasminogen activator urokinase receptor (PLAUR) (10 incident outcomes). These markers of multiple morbidities were also identified in logistic regression models run between the protein levels and multimorbidity status (Supplementary Table 7 and Supplementary Note).

A sensitivity analysis modeled each of the 35,232 Cox PH associations tested over increasing yearly case follow-up intervals. Of the 3,209 associations, 2,915 and 1,957 had *P* < 3.1 × 10^−6^ (the Bonferroni-adjusted threshold) when restricting cases up to 10- and 5-year onset, respectively (Supplementary Tables 8 and 9 and Supplementary Note). These results can be examined in a Shiny app available at https://protein-disease-ukb.optima-health.technology. The app also includes an interactive network of the 3,209 associations.

A second sensitivity analysis explored the potential impact of medication use in a subset of the population that had this information available (35,073 individuals). Ischemic heart disease was chosen given that a range of blood pressure-lowering medications are commonly used to delay or prevent this disease. Of the 371 protein–ischemic heart disease associations that had *P* < 3.1 × 10^−6^ in the fully adjusted models in this subset, 336 remained statistically significant at the same *P* value threshold after adjusting for the use of blood pressure-lowering medications at baseline (Supplementary Table 10 and Supplementary Note).

### ProteinScore development

**Extended Data Fig. 3:**
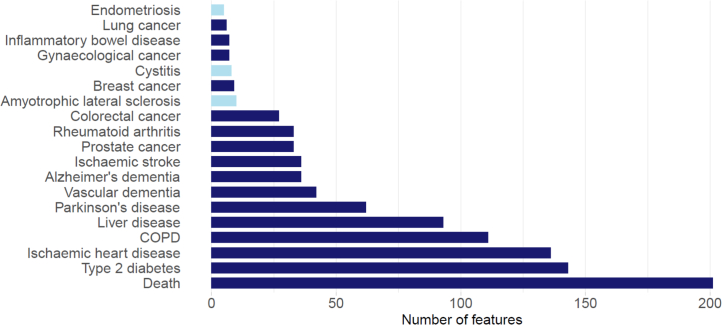
ProteinScore feature selection. The total number of contributing protein analyte features selected for each ProteinScore. Incident outcomes that were assessed for 5-year onset (light blue) and 10-year onset (dark blue) are delineated. Source data

**Extended Data Fig. 4:**
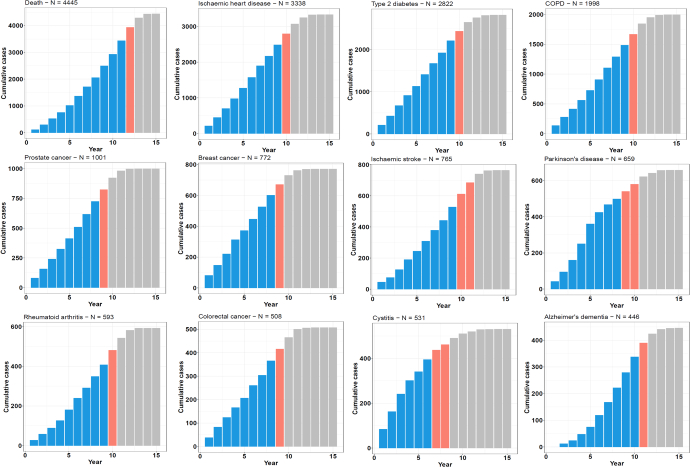
Cumulative time-to-onset for cases by outcome in the UK Biobank PPP sample. Case counts are shown for each trait, with the number of cases by year of follow-up plotted cumulatively and the year that the proportion of cases diagnosed reached 80% (orange) and 90% (grey) demarcated. COPD: chronic obstructive pulmonary disease. Source data

**Extended Data Fig. 5:**
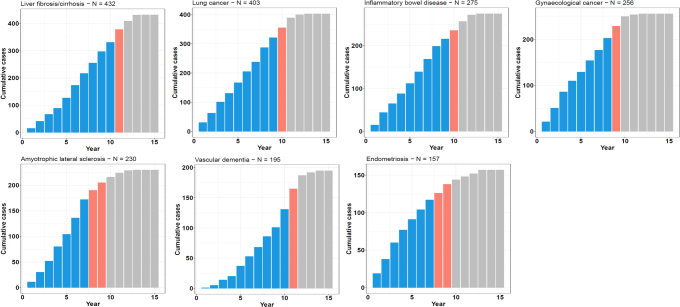
Cumulative time-to-onset for cases by outcome in the UK Biobank PPP sample. Case counts are shown for each trait, with the number of cases by year of follow-up plotted cumulatively and the year that the proportion of cases diagnosed reached 80% (orange) and 90% (grey) demarcated. Source data

We developed ProteinScores by Cox PH elastic net regression for 19 diseases that had a minimum of 150 incident cases. Of 50 randomized iterations (Methods), ProteinScores with the median difference in the area under the curve (AUC) beyond a minimally adjusted model were selected for each outcome (Supplementary Table 11). Summaries of protein features for the 19 ProteinScores are available in Supplementary Tables 12 and 13, ranging from 5 features for endometriosis to 201 features for all-cause mortality (Extended Data Fig. 3). Cumulative time-to-onset distributions for cases (Extended Data Figs. 4 and 5) indicated that amyotrophic lateral sclerosis, endometriosis and cystitis were better suited to 5-year-onset assessments (80% of cases diagnosed by year 8 of follow-up). All remaining ProteinScores were evaluated for 10-year onset.

**Fig. 2:**
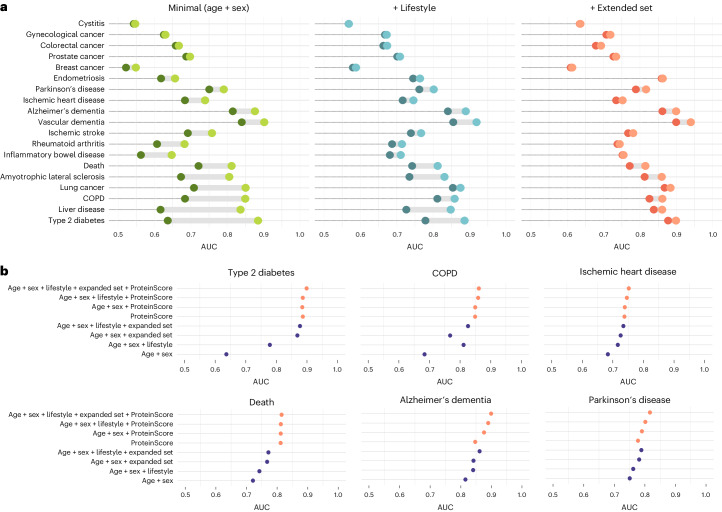
Value offered by ProteinScores for incident outcomes in the UK Biobank. **a**, Differences in AUC resulting from the addition of the 19 ProteinScores to models with increasingly extensive sets of covariates: minimally adjusted (age and sex in which traits were not sex-stratified) in green, minimally adjusted with the addition of a core set of six lifestyle covariates in blue, and further adjustment for an extended set of 18 covariates that are measured in clinical settings (physical and biochemical measures) in orange. AUC plots are ordered by increasing AUC differences in the minimally adjusted models. All ProteinScore performance statistics shown correspond to 10-year onset, except those for amyotrophic lateral sclerosis, endometriosis and cystitis, which were assessed for 5-year onset. Darker-shaded points indicate the base covariate model used, whereas lighter-shaded points connected by gray shading indicate the difference added by the addition of the ProteinScore into the model. **b**, A breakdown of the AUC values achieved by different combinations of risk factors with and without the ProteinScores is shown for the six incident outcomes whereby the ProteinScore contributed statistically significantly beyond a Cox PH model including all 24 minimal, lifestyle and extended set variables (ROC *P* < 0.0026, the Bonferroni-adjusted threshold). All six of the best-performing ProteinScores shown were assessed for the 10-year onset of the disease. Results that include the ProteinScore are shaded in orange, whereas results that do not are shaded in purple. Two-sided tests were used in all cases. Source data

**Extended Data Fig. 6:**
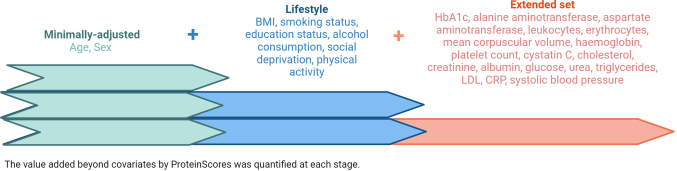
Comprehensive covariates that were modelled to evaluate the value added by the ProteinScores beyond these covariates. Three increasingly complex sets of covariates were considered: 1) age and sex (where traits had not been sex-stratified), 2) further adjustment for a core set of six lifestyle and health covariates (BMI, alcohol consumption, social deprivation, educational attainment, smoking status and physical activity) and 3) further adjustment for an extended set of 18 biochemistry and physical attributes that are measurable in clinical settings. Performance when using only the ProteinScores was also considered. When modelled alongside age and sex, 26 possible covariates were therefore used in maximally-adjusted models. Created with BioRender.com.

Selected ProteinScores were modeled alongside combinations of covariates (Extended Data Fig. 6). The differences in AUC resulting from the addition of the ProteinScores into the three models with increasingly complex sets of covariates are summarized in Fig. 2a. A tabular summary of the AUC statistics is available in Supplementary Table 14. Singular inclusion of the ProteinScores had either equal or higher performance than the maximal set of 26 covariates in eight instances. Tests for significant differences between receiver operating characteristic (ROC) curves for the sets of covariates with and without the ProteinScores were performed. Eleven ProteinScores had ROC *P* < 0.0026 (the Bonferroni-adjusted *P* value threshold) beyond minimally adjusted covariates. When ProteinScores were added to models that included both minimally adjusted and lifestyle covariates, nine ProteinScores had *P* < 0.0026 in ROC model comparison tests. When ProteinScores were added to models that further adjusted for an additional 18 clinically measurable covariates, six ProteinScores (type 2 diabetes, chronic obstructive pulmonary disease (COPD), death, Alzheimer’s dementia, ischemic heart disease and Parkinson’s disease) had *P* < 0.0026 in model comparisons with and without the ProteinScore (Fig. 2b).

### Exploration of the type 2 diabetes ProteinScore

**Fig. 3:**
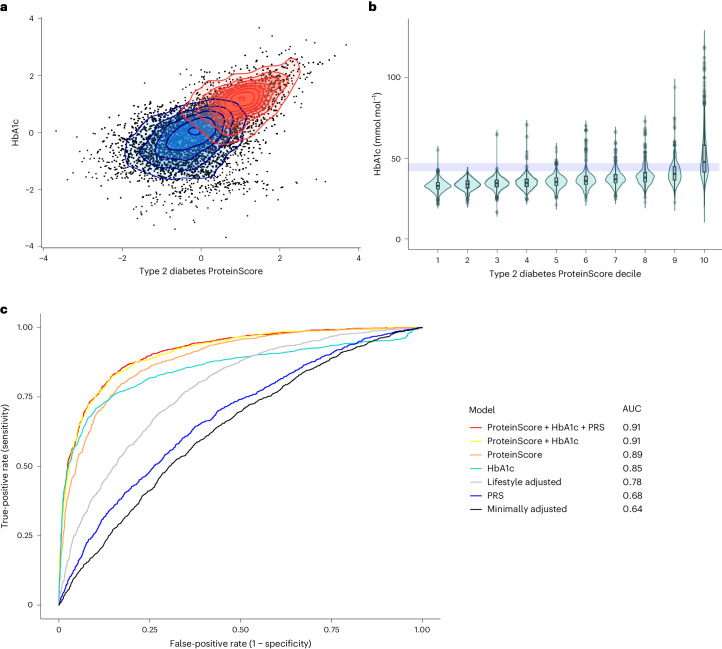
Exploration of the type 2 diabetes ProteinScore. **a**, Case (red) and control (blue) discrimination for HbA1c and the type 2 diabetes ProteinScore in the test set (1,105 cases and 3,264 controls, mean time to case onset 5.4 years (s.d. 3.0 years)). Both markers were rank-based inverse normalized and scaled to have a mean of 0 and s.d. of 1. **b**, HbA1c (mmol mol^−1^) per decile of the type 2 diabetes ProteinScore in the test set (1,105 cases and 3,264 controls, mean time to case onset 5.4 years (s.d. 3.0 years)). The shaded rectangle indicates the type 2 diabetes HbA1c screening threshold (42–47 mmol mol^−1^). Violin plots display the median and upper and lower quartiles as the three lines comprising the central rectangle, with minima and maxima points corresponding to those at the tips of the plot whiskers. **c**, ROC curves for incremental 10-year-onset models incorporating HbA1c, the type 2 diabetes ProteinScore and a PRS for type 2 diabetes individually and concurrently. Source data

Type 2 diabetes was chosen as a case study for exploration. Glycated hemoglobin (HbA1c) averages long-term glucose levels over 2–3 months and is used to monitor preclinical diabetes risk (42–47 mmol mol^−1^) and to diagnose the disease (with two repeated measurements >48 mmol mol^−1^)^26,27^. As the ProteinScore for type 2 diabetes added value beyond the extended set of covariates that included HbA1c, we directly compared the performance of HbA1c and the ProteinScore in the test sample alongside a PRS for type 2 diabetes. In the test set, 1,105 cases (mean time-to-onset 5.4 years (s.d. 3.0 years)) and 3,264 controls had all measures available. The rank-based inverse normal transformed levels of the ProteinScore and HbA1c discriminated incident case and control distributions similarly (Fig. 3a), and HbA1c levels tended to be higher across ProteinScore risk deciles (Fig. 3b). In incremental Cox PH models for the 10-year onset of type 2 diabetes (Fig. 3c), the singular use of the ProteinScore (AUC = 0.89) outperformed both HbA1c (AUC = 0.85) and the PRS (AUC = 0.68). In ROC model comparisons between HbA1c alone and HbA1c with the ProteinScore, a significant improvement due to the ProteinScore was identified (ROC *P* < 0.0026). When the PRS was added to this model (including HbA1c and the ProteinScore), the AUC remained unchanged (0.91) (Supplementary Table 15).

### Metabolomic score comparison

**Extended Data Fig. 7:**
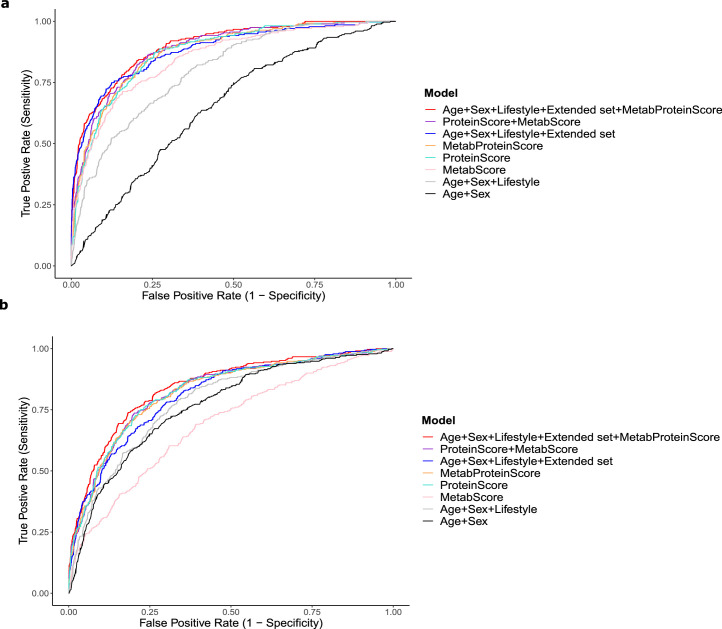
Comparison of metabolomic and proteomic feature performance for type 2 diabetes and all-cause mortality traits. ROC curves for 10-year onset scores developed in the subsets of the training and test populations that had metabolomics and proteomics available. A Metabolomic score (MetaboScore), ProteinScore and a joint omics score (MetaboProteinScore) are modelled individually and concurrently and benchmarked against either age and sex, six lifestyle factors, or an ‘extended set’ including these variables in addition to a further 18 clinically relevant covariates. **a**, ROC curve comparison for type 2 diabetes. **b**, ROC curve comparison for all-cause mortality. Full summary statistics are available in Supplementary Table 16. Source data

In a sensitivity analysis, we considered metabolomic and proteomic features for score generation. Type 2 diabetes and all-cause mortality were chosen for the following reasons: (1) they had a large number of cases; (2) ProteinScores for these traits were among the top-performing ProteinScores; and (3) there is evidence that both traits may be stratified by metabolomic features^15,21^. A total of 12,050 of the 47,600 individuals with protein data had metabolomics data (Supplementary Note). Test sets used for ProteinScores were subset to those with metabolomics data, for type 2 diabetes (*n* cases_train_ = 377, *n* controls_train_ = 1,002, *n* cases_test_ = 309, *n* controls_test_ = 898) and mortality (*n* cases_train_ = 616, *n* controls_train_ = 1,680, *n* cases_test_ = 410, *n* controls_test_ = 1,048). The performance of a MetaboScore (considering metabolite features), ProteinScore (considering protein features) and MetaboProteinScore (considering combined metabolomic and proteomic features) is summarized for both traits in Extended Data Fig. 7 and Supplementary Table 16. The selected features are available in Supplementary Table 17. For all-cause mortality, the ProteinScore (AUC = 0.82) outperformed the MetaboScore (AUC = 0.69), with an AUC of 0.83 when both individual scores were modeled concurrently. For type 2 diabetes, the ProteinScore (AUC = 0.87) and MetaboScore (AUC = 0.85) were more comparable in performance, with an additive AUC of 0.89 when both individual scores were modeled concurrently.

## Discussion

This study quantified circulating proteome signatures that are reflective of multiple incident diseases in mid to later life. These data suggest that augmenting traditional risk factors with proteomic, metabolomic and genetic data types may further hone risk stratification.

We demonstrated that relatively few circulating proteins can add value to risk stratification up to a decade before formal diagnoses. ProteinScores for incident type 2 diabetes, COPD, ischemic heart disease, Alzheimer’s dementia, Parkinson’s disease and death demonstrated value beyond a comprehensive set of 26 covariates; equal or higher AUCs were observed for models including all covariates compared to those with only the ProteinScore. This suggests that ProteinScores can absorb a large proportion, if not all, of the typical covariate signal. The scores minimize the need for the extensive recording of lifestyle, physical and biomarker measures, offering a streamlined set of metrics to proxy for an individual’s health status.

While much interest is currently devoted to using PRS for disease prediction, these scores neglect environmental components of disease risk and may, therefore, be limited in the context of complex age-related diseases^28,29^. Our ProteinScore for type 2 diabetes outperformed the PRS, likely due to proteins representing an interface that captures genetic, environmental and lifestyle contributions to disease risk. The improvement in AUC resulting from concurrent modeling of HbA1c and the type 2 diabetes ProteinScore suggests that the latter provides additional value.

Our results suggest that jointly considering ProteinScores with scores generated using metabolomic features may further augment risk stratification. An additive improvement resulting from the addition of the MetaboScore to the ProteinScore model was observed for all-cause mortality and type 2 diabetes. However, the ProteinScores tended to outperform the MetaboScores, particularly with respect to the results for all-cause mortality. For type 2 diabetes, the comparable performance of the MetaboScore to the ProteinScore (AUCs of 0.85 and 0.87, respectively) was impressive given the limited number of input features available from the metabolomic assay (249 potential features, of which 81 were ratios between metabolites). These examples highlight the need for scoring assessments on a disease-by-disease basis, as it is likely that some omics types will be more suited to certain diseases. Joint consideration of protein and metabolite measures in the full UK Biobank cohort would hold promise to resolve these signatures further. Similarly, integration of additional omics types such as DNA methylation—known to track lifestyle traits, biological aging states and disease risk^30–32^—would also be recommended if these data were available. For metabolomic stratification of incident mortality, we emphasize that the MetaboHealth score is the current best-performing and preferred metric, trained on a larger sample than ours (5,512 versus 616 deaths)^15^.

A subset of the individual protein–disease associations we report likely represents direct mediators of disease. We encourage exploring this further through techniques such as Mendelian randomization and colocalization. Modeling that considers multimorbidity trajectories over the life course would aid in understanding the role of prevalent diseases and medication use in future disease risk. The largest number of associations and the strongest effect sizes (by the magnitude of the absolute log of the HR) were observed for liver disease. For neurological diseases and cancers, where fewer associations were identified, it is possible that bulk blood is less able to capture the full spectrum of disease pathogenesis, which may be localized to distal or more refined tissues. Similarly, the panel of proteins available may reflect certain diseases better than others. Despite having relatively few individual protein associations, the Alzheimer’s dementia ProteinScore was one of the best-performing ProteinScores and was largely unchanged upon the addition of covariates. As therapeutic interventions for neurodegenerative diseases have greater efficacy when implemented earlier in the disease pathogenesis^33–35^, ProteinScores such as this may help with trial recruitment. Correlations between the covariates and ProteinScores (Supplementary Table 18) suggest that the former reflect a range of lifestyle, physiological and health measures, indicating that they may be useful measures to proxy for health status.

Of the 720 proteins that were identified as indicators of multimorbidity status, 716 were associated with age (Bonferroni-adjusted *P* < 1.7 × 10^−5^, with 648 having positive effect sizes) in a previous analysis of the same dataset (Supplementary Table 5 in ref. ^22^). Future studies could explore their possible causal contributions to disease and whether they have differential effects across the life course. Examples of such proteins include GDF15, IL-6 and PLAUR—three proteins that had the largest number of associations with individual incident diseases in our study. GDF15 was previously identified as the top marker of future multimorbidity from 1,301 plasma proteins tested^36,37^. IL-6 mediates chronic, low-grade inflammation and is a key biomarker of aging^38^, with anti-IL-6 antibodies developed for a range of inflammation-associated diseases^39,40^. PLAUR has previously been associated with incident cancer, cardiovascular disease and diabetes^41^.

This study has several limitations. First, the assessment of scores by regression within a test sample, followed by the calculation of an AUC, is not a direct prediction and cannot translate easily to new populations. Second, nonrandom selection of disease cases through the UKB-PPP consortium may have introduced biases. The UK Biobank study may also be prone to selection bias, as the individuals recruited may represent those who have better health than the general population. Third, it was not possible to source an external test set for the ProteinScores with sufficient incident case counts to enable a meaningful replication assessment. Fourth, variation in protein analyte levels across measurement technologies has been reported^42^. Fifth, the proteins measured were recorded on a relative scale, which limits the translation of scores to new populations. Sixth, death was treated as a censoring event; competing risks and multistate modeling approaches may provide a more nuanced analytical strategy. Finally, the UK Biobank population is largely composed of individuals with European, white British ancestry and a restricted age range (40–71 years, with a mean of 57 years), which may limit the generalizability of the findings. Future studies in equally well-characterized cohorts will be needed to assess translation to other populations, age ranges and ethnicities.

## Methods

### The UK Biobank sample population

The UK Biobank is a population-based cohort of approximately 500,000 individuals aged between 40 and 69 years who were recruited between 2006 and 2010. Data from genome-wide genotyping, exome sequencing, electronic health record linkage, whole-body magnetic resonance imaging, blood and urine biomarker assays, and physical and anthropometric measurements are available. More information regarding the full measurements can be found at https://biobank.ndph.ox.ac.uk/showcase/. The UKB-PPP is a precompetitive consortium of 13 biopharmaceutical companies funding the generation of blood-based proteomic data from UK Biobank volunteer samples. This research has been conducted using the UK Biobank resource under approved application numbers 65851, 20361, 26041, 44257, 53639 and 69804. All participants provided informed consent.

### Proteomics in the UK Biobank

The UKB-PPP sample includes 54,219 UK Biobank participants and 1,474 protein analytes measured across four Olink panels (cardiometabolic, inflammation, neurology and oncology; annotation information is provided in Supplementary Table 1)^22^. A randomized subset of 46,595 individuals was selected from the baseline UK Biobank cohort, with 6,376 individuals selected by members of the UKB-PPP consortium and 1,268 individuals included who participated in a COVID-19 study. The randomized samples have been shown to be highly representative of the wider UK Biobank population, whereas the consortium-selected individuals were enriched for 122 diseases^22^. Details on sample selection for the UKB-PPP are provided in the Supplementary Note. Of 54,219 individuals who had protein data, 52,744 were available after quality control exclusions (as per ref. ^22^), with 1,474 Olink protein analytes measured (annotations in Supplementary Table 1)^22^. The maximum sample size possible was therefore taken forward for the study. The sample is predominantly white/European (93%) but also includes individuals with Black/Black British, Asian/Asian British, Chinese, mixed, other and missing ethnic backgrounds (7%). The study by Sun et al.^22^ includes associations between the protein levels studied here and age, sex, lifestyle and health factors. Data collection and analysis were not performed blind to the conditions of the experiments.

Extended Data Fig. 2 summarizes the processing steps applied to this dataset to derive a complete set of measurements for use. Briefly, of 107,161 related pairs of individuals (calculated through kinship coefficients >0 across the full UK Biobank cohort), 1,276 pairs were present in the 52,744 individuals. After the exclusion of 104 individuals in multiple related pairs, in addition to 1 individual randomly selected from each of the remaining pairs, there were 51,562 individuals. A further 3,962 individuals were excluded because of having >10% missing protein measurements. Four proteins that had >10% missing measurements (CTSS.P25774.OID21056.v1 and NPM1.P06748.OID20961.v1 from the neurology panel, PCOLCE.Q15113.OID20384.v1 from the cardiometabolic panel and TACSTD2.P09758.OID21447.v1 from the oncology panel) were then excluded. The remaining 1% of missing protein measurements were imputed by *k*-nearest-neighbor (*k* = 10) imputation using the impute R package (version 1.60.0)^43^. The final dataset consisted of 47,600 individuals and 1,468 protein analytes. Assessments of the protein batch, study center and genetic principal components suggested that these factors had minimal effects on protein levels (lowest correlation between protein levels and residuals of 0.94) (Supplementary Note). Therefore, protein levels were not adjusted for these factors.

### Phenotypes in the UK Biobank

Demographic and phenotypic information for the 47,600 individuals with complete protein data for 1,468 analytes is available in Supplementary Table 2. Lifestyle covariates included BMI (weight in kilograms divided by height in meters squared), alcohol intake frequency (1 = daily or almost daily, 2 = three to four times a week, 3 = once or twice a week, 4 = one to three times a month, 5 = special occasions only, 6 = never), the Townsend index of deprivation (higher score representing greater levels of deprivation) and smoking status (0 = never, 1 = previous, 2 = current), physical activity (0 = between 0 and 2 days per week of moderate physical activity, 1 = between 3 and 4 days per week of moderate physical activity, 2 = between 5 and 7 days per week of moderate physical activity) and education status (1 = college/university educated, 0 = all other education). Of the 47,600 individuals with complete protein data, there were 52, 52, 236, 56 and 59 missing entries for alcohol, smoking, BMI, physical activity and deprivation, respectively. No imputation of missing data was performed for the inclusion of these variables in individual Cox PH analyses. There were an additional 2,556, 188 and 59 individuals who responded with ‘prefer not to answer’ and were excluded from physical activity, smoking and alcohol variables, respectively.

### Electronic health data linkage in the UK Biobank

Electronic health linkage to National Health Service records was used to collate incident diagnoses. Death information was sourced from the death registry data available through the UK Biobank. Cancer outcomes were sourced from the cancer registry (International Classification of Diseases (ICD) codes), whereas noncancer diseases were sourced from first-occurrence traits available in the UK Biobank. The first-occurrence traits integrate general practice (Read2/3) ICD (version 9/10) data with self-report and ICD codes present on the death registry to identify the earliest date of diagnosis. These data sources are linked to three-digit ICD trait codes. The following 23 diseases were included: liver disease, systemic lupus erythematosus, type 2 diabetes, amyotrophic lateral sclerosis, Alzheimer’s dementia, endometriosis, COPD, inflammatory bowel disease, rheumatoid arthritis, ischemic stroke, Parkinson’s disease, vascular dementia, ischemic heart disease, major depressive disorder, schizophrenia, multiple sclerosis, cystitis, and lung, prostate, breast, gynecological, brain/central nervous system and colorectal cancers. These represent a selection of leading age-related causes of morbidity, mortality and disability. In all analyses involving sex-specific diseases, the population was stratified into male and female groups, and sex was not included as a covariate in incremental Cox PH assessments. Traits that were stratified included gynecological cancer, breast cancer, endometriosis and cystitis (all female-stratified) and prostate cancer (male-stratified).

The date of diagnosis for each disease was ascertained through electronic health linkage. Based on the date of baseline appointment, the time to first onset for each diagnosis was calculated in years. For controls, time to onset was defined as the time from baseline to the censoring date. Death was treated as a censoring event. Time to censor date was calculated for the controls who remained alive. In contrast, if a control individual had died during the follow-up, time to death was taken forward for Cox PH models. Any cases that were prevalent at baseline were excluded. Alzheimer’s and vascular dementias were restricted to an age at onset (or censoring) of 65 years or older in all analyses. Sex-specific traits were stratified across all analyses.

### Statistics and reproducibility

Cox PH models were run between each protein and each incident disease using the ‘survival’ package (version 3.4-0)^44^ in R (version 4.2.0)^45^. Protein levels were rank-based inverse normalized and scaled to have a mean of 0 and s.d. of 1 before analyses. Minimally adjusted Cox PH models for sex-stratified traits included age at baseline as a covariate, whereas the remaining models adjusted for age and sex. Lifestyle-adjusted models further controlled for education status, BMI, smoking status, social deprivation rank, physical activity and alcohol intake frequency. A Bonferroni-adjusted *P* value threshold for multiple testing based on the 678 components that explained 90% of the cumulative variance in the 1,468 protein analyte levels (Supplementary Table 3) and 24 outcomes tested was applied across all Cox PH models (*P* < 0.05/(678 × 24) = 3.1 × 10^−6^ was used as the Bonferroni-adjusted *P* value threshold). PH assumptions were checked by examining protein-level Schoenfeld residuals.

A sensitivity analysis was performed for each of the 35,232 fully adjusted associations tested, restricting cases to successive years of follow-up. These sensitivity analyses were visualized using the Shiny package (version 1.7.3)^46^ in R. The magnitude of the change in HR for individual associations can be examined by the year of case follow-up to assess the consistency of effect sizes. A network visualization was also created within the Shiny interface to highlight the fully adjusted associations that had *P* < 3.1 × 10^−6^ using the networkD3 (version 3.0.4)^47^ and igraph (version 1.3.5)^48^ R packages. To verify further the markers of multiple morbidities identified in individual Cox PH analyses, we also run logistic regression models between each of the 1,468 protein analyte levels and multimorbidity status (defined as 1,454 individuals who received three or more of the 23 disease diagnoses over the 15-year follow-up period). A sensitivity analysis was also done for ischemic heart disease associations with and without adjustment for blood pressure-lowering medications reported at baseline in a subset of individuals (35,073 of 47,600) who had medication information available. The Supplementary Note provides details on the classification of medications as per the anatomical therapeutic chemical classification categories. A total of 14,074 individuals (of the 35,073) indicated that they were taking one or more blood pressure-lowering medications at baseline. This was treated as a binary variable, and the comparison with and without adjustment for this variable was performed for ischemic heart disease Cox PH associations in the subset of 35,073 individuals. Adjustments for age, sex and six lifestyle factors were included in both sets of analyses, with 2,456 cases and 27,468 controls.

**Extended Data Fig. 8:**
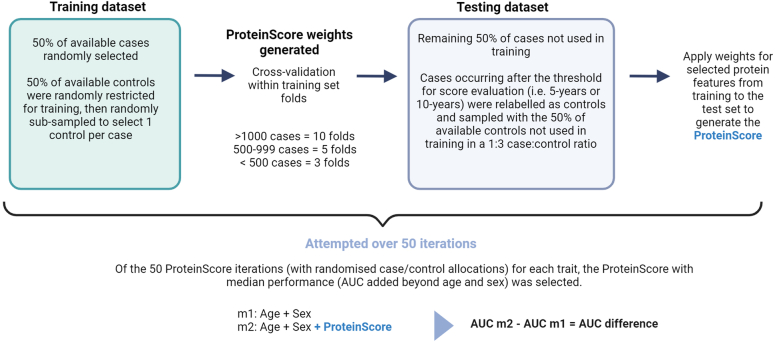
Summary of the ProteinScore development pipeline. ProteinScores were developed across fifty randomised iterations. For each iteration, 50% of available cases were randomly allocated to the training set and 50% of controls were randomly sampled to obtain a 1:3 case:control ratio. Cox PH elastic net regression with cross-fold validation across folds of the training sample was used to derive weighting coefficients. The 50% of cases that were not included in the training set were allocated to the test set. If cases in the test set occurred after the threshold for onset evaluation (that is 5-year or 10-year), they were relabelled as controls and randomly sampled with the 50% of controls not considered during training, to obtain a 1:3 case:control ratio. Of the fifty ProteinScore iterations tested, the ProteinScore that yielded the median incremental difference to the Area Under the Curve (AUC) beyond a minimally-adjusted model was identified. If no features were selected for an iteration, it was weighted with a performance of 0 in median AUC selection. If features were selected for an iteration but the randomly sampled test set included no cases at or beyond the onset threshold (precluding extraction of baseline hazard at this point for AUC calculation) these models were excluded from the median ProteinScore selection. Created with BioRender.com.

MethylPipeR^32^ is an R package with an accompanying user interface that we have previously developed for the systematic and reproducible development of incident disease predictors. Using MethylPipeR, we trained ProteinScores that considered 1,468 Olink protein levels by Cox PH elastic net regression through the R package ‘glmnet’ (version 4.1-4)^49^. Penalized regression minimizes overfitting by using a regularization penalty, and the best shrinkage parameter (*λ*) was chosen by cross-fold validation with *α* fixed to 0.5. Of the 24 outcomes featured in the individual Cox PH analyses, 19 that had a minimum case count of 150 were selected for ProteinScore development. The chosen strategy for ProteinScore development included training ProteinScores for each trait across 50 randomized iterations (with each iteration including a different combination of cases and controls in the train and test sets). Random assignment was determined through random sampling across a list of sample identifier numbers pertaining to study individuals in R (version 4.2.0)^45^. This strategy quantifies the stability of the ProteinScore performance, which is critical given that unobserved confounders may be enriched during the random selection of individuals from the wider population. The ProteinScore training strategy is summarized in Extended Data Fig. 8. Briefly, 50 iterations of each ProteinScore were performed that randomized sample selection by 50 randomly sampled seeds (values between 1 and 5,000). For each iteration, cases and controls were randomly split into 50% groups for training and testing. From the 50% training control population, a subset of controls was then randomly sampled to give a case-to-control ratio of 1:3 to balance the datasets. For traits with >1,000 cases in training samples, ten folds were used. For traits with between 500 and 1,000 cases in training, five folds were used. Three folds were used when there were <500 cases in the training sample. Protein levels were rank-based inverse normalized and scaled to have a mean of 0 and s.d. of 1 in the training set.

Cumulative time-to-onset distributions for cases (Extended Data Figs. 4 and 5) indicated that amyotrophic lateral sclerosis, endometriosis and cystitis were better suited to 5-year-onset assessments in the test sample (80% of cases were diagnosed at 8 years after baseline). All remaining ProteinScores were tested in the context of 10-year onset (80% of cases were not diagnosed 8 years after baseline). Across the 50 ProteinScore iterations for each trait, 50% of cases and controls that were not randomly selected for training were reserved for testing. For a visualization of the test set sampling and assessment strategy, see Extended Data Fig. 8. In the test set, cases that had time to event up to or including the 5- or 10-year threshold used for onset prediction were selected, whereas cases beyond the threshold were placed with the control population, which was then randomly sampled in a 1:3 ratio. Weighting coefficients for features selected during ProteinScore training were used to project scores into the test sample. Incremental Cox PH models were run in the test sample to obtain cumulative baseline hazard and onset probabilities, which were used to derive AUC estimates. The test set sampling strategy ensured that, while most cases occurred up to the onset threshold, a small proportion (~3%) of cases were included in Cox PH models with onset times after the 10- or 5-year threshold to simulate a real-world scenario for risk stratification. If cases fell beyond the 5- or 10-year threshold for onset, they were recoded as controls in the AUC calculation. Cumulative baseline hazard probabilities were calculated using the Breslow estimator available in the ‘gbm’ R package (version 2.1.8.1)^50^. Survival probabilities were then generated by taking the exponential of the negative cumulative baseline hazard at 5 or 10 years to the power of the Cox PH prediction probabilities. ProteinScore onset probabilities were calculated as 1 minus these survival probabilities. AUC and ROC statistics were extracted for the survival probabilities using the calibration function from the ‘caret’ R package (version 6.0-94)^51^ and the evalmod function from the ‘MLmetrics’ R package (version 1.1.1)^52^.

ProteinScores that yielded the median incremental difference to the AUC of a minimally adjusted model (adjusting for age or age and sex) were selected from the 50 possible ProteinScores for each trait. If no features were selected during training, models were weighted as a performance of 0 in the median model selection. In some instances, features were selected during training and incremental Cox PH models were run successfully, but the random sampling of the test set did not include a case with time to event at or after the 5- or 10-year onset threshold. Therefore, these models were excluded as cumulative baseline hazard distributions did not reach the onset threshold and could not be extracted for AUC calculations. The number of models with minimum and maximum performance was documented (Supplementary Table 11). This approach mitigated the presence of extreme case–control profiles driving ProteinScore performance and minimized the possibility of bias being introduced by selecting train and test samples based on matching for specific population characteristics.

Selected ProteinScores for each trait were then evaluated to quantify the additional value (in terms of increases in AUC) that resulted from the addition of ProteinScores. Minimally adjusted models included age and sex (if traits were not sex-stratified). Lifestyle-adjusted models then further accounted for common lifestyle covariates (education status, BMI, smoking status, social deprivation rank, physical activity and alcohol intake frequency). Finally, models including covariates from the minimally adjusted, lifestyle-adjusted and an extended set of clinically measured variables were then assessed (Extended Data Fig. 6). In each case, the difference in AUC resulting from the addition of the ProteinScore was reported. ROC *P* value tests were used to ascertain whether the improvements offered by selected ProteinScores for each outcome were statistically significant, beyond each set of increasingly saturated covariates. A Bonferroni-adjusted *P* value threshold for ROC *P* tests was used based on the 19 ProteinScore traits (*P* < 0.05/19 = 0.0026). The ‘precrec’ R package (version 0.12.9)^53^ was used to generate ROC and precision–recall curves for each ProteinScore.

A set of 26 possible covariates used across the minimally adjusted, lifestyle-adjusted and extended set analyses were assessed for missingness, imputed (where missingness was <10%) and used in the ProteinScore evaluation as a maximal, extended set of covariates. Further details on variable selection and preparation are supplied in the Supplementary Note. Additional covariates (considered in addition to age, sex and the six lifestyle traits used in individual Cox PH analyses) included leukocyte counts (10^9^ cells per liter), erythrocyte counts (10^12^ cells per liter), hemoglobin concentration (g dl^−1^), mean corpuscular volume (fl), platelet count (10^9^ cells per liter), cystatin C (mg l^−1^), cholesterol (mmol l^−1^), alanine aminotransferase (U l^−1^), creatinine (μmol l^−1^), urea (mmol l^−1^), triglycerides (mmol l^−1^), low-density lipoprotein (mmol l^−1^), C-reactive protein (mg l^−1^), aspartate aminotransferase (U l^−1^), HbA1c (mmol mol^−1^), albumin (g l^−1^), glucose (mmol l^−1^) and systolic blood pressure (mm Hg). After the covariate processing steps were complete, a population of 43,437 individuals was available with complete information for ProteinScore testing. Phenotypic summaries of the additional covariates for this population are provided in Supplementary Table 2.

### Further assessment of the type 2 diabetes ProteinScore

HbA1c is a blood-based measure of chronic glycemia that is highly predictive of type 2 diabetes events and is recommended as a test of choice for the monitoring and diagnosis of type 2 diabetes^26,27^. HbA1c (mmol mol^−1^) measurements (field ID 30750) and the type 2 diabetes PRS available in the UK Biobank (field ID 26285) were extracted. A contour plot showing both variables grouped by those who went on to be diagnosed with type 2 diabetes over a 10-year period was created. HbA1c levels were also plotted against ProteinScore risk deciles. HbA1c and the ProteinScore levels were rank-based inverse normalized and assessed individually and concurrently in incremental models for the 10-year onset of type 2 diabetes in the ProteinScore test set. The 10-year incremental Cox PH models were used to derive onset probabilities for the calculation of AUCs after adding the ProteinScore to models adjusting for HbA1c and the type 2 diabetes PRS. Model comparisons were used (test of the difference in ROC curves) to quantify the value added by the ProteinScore beyond the PRS and HbA1c.

### Preliminary metabolomics assessment

Metabolomics measures were available for 12,050 of the 47,600 individuals with proteomic data included in the study (see the Supplementary Note for details on data preparation). Type 2 diabetes and death were chosen as case studies for further exploration. The train and test sets used to develop the main ProteinScores were subset to those with metabolomics data available for type 2 diabetes (*n* cases_train_ = 377, *n* controls_train_ = 1,002, *n* cases_test_ = 309, *n* controls_test_ = 898) and death (*n* cases_train_ = 616, *n* controls_train_ = 1,680, *n* cases_test_ = 410, *n* controls_test_ = 1,048). Scores that considered only metabolomic features (MetaboScore), only proteomic features (ProteinScore) and joint omics features (MetaboProteinScore) were trained and tested in these populations. There were 249 metabolite measures (comprising 168 metabolites and 81 ratios between combinations of metabolites) and 1,468 protein levels considered as potentially informative features. Performance was evaluated for the 10-year onset of type 2 diabetes and death in the test sample, modeling scores individually and concurrently and benchmarking them against the maximal set of 26 possible covariates (Extended Data Fig. 6).

### Reporting summary

Further information on research design is available in the Nature Portfolio Reporting Summary linked to this article.

### Supplementary information

Supplementary Information Supplementary Note.

Reporting Summary

Supplementary Tables 1–18 Supplementary Table 1. Protein annotations for the 1,472 Olink analytes available in the UK Biobank PPP sample. Supplementary Table 2. Demographic and phenotypic information for the 47,600 individuals with complete protein information used in the analyses. Supplementary Table 3. Principal component analyses for the 1,468 protein analytes used in the study. Supplementary Table 4. Minimally adjusted Cox PH associations between protein analytes and 24 incident outcomes in the UK Biobank PPP sample. Supplementary Table 5. Retained Cox PH associations between protein analytes and 24 incident outcomes in the UK Biobank PPP sample after adjustment for age, sex and a further six lifestyle covariates. Supplementary Table 6. Summary of protein analytes that were associated with incident outcomes in the UK Biobank PPP sample (*n* = 47,600). Supplementary Table 7. Protein analyte associations with multimorbidity status (binary trait, based on having three or more morbidities diagnosed over the 16-year follow-up period). Supplementary Table 8. Cox PH sensitivity analyses: restricting case follow-up to successive yearly intervals. Supplementary Table 9. Summary table for the Cox PH sensitivity analyses: restricting case follow-up to 10- and 5-year onset. Supplementary Table 10. Sensitivity analyses for ischemic heart disease with and without adjustment for blood pressure-lowering medication use. Supplementary Table 11. Median ProteinScore selection from 50 randomly sampled iterations. Supplementary Table 12. Coefficient weights for protein analytes selected as informative features for ProteinScores in UK Biobank PPP. Supplementary Table 13. Proteins selected as informative features for 19 ProteinScores developed in the UK Biobank PPP sample. Supplementary Table 14. Performance of the 19 ProteinScores in the test sample. Supplementary Table 15. Type 2 diabetes ProteinScore performance against HbA1c and a polygenic risk score (PRS) in 10-year Cox PH models in the test sample. Supplementary Table 16. Preliminary analyses comparing metabolomics and proteomics data sources in a subset of the type 2 diabetes and death test samples with both omics measures available. Supplementary Table 17. Features selected for metabolomic, proteomic and joint (metabolomic and proteomic) scores. Supplementary Table 18. Correlations between ProteinScores in the respective test sets and a range of lifestyle and clinical measures included in the extended set.

### Source data

Source Data Fig. 1 Underlying data used to generate the plots.

Source Data Fig. 2 Underlying data used to generate the plots.

Source Data Fig. 3 Underlying data used to generate the plot in Fig. 3c.

Source Data Extended Data Fig. 3 Underlying data used to generate the plot.

Source Data Extended Data Figs. 4 and 5 Underlying data used to generate the plots.

Source Data Extended Data Fig. 7 Underlying data used to generate the plots.

## Data Availability

Datasets generated in this study are made available in the supplementary tables. Proteomics data are available as part of the UK Biobank. The data can be accessed through the UK Biobank Research Analysis Portal (https://www.ukbiobank.ac.uk/enable-your-research). In the portal, the UK Biobank has cataloged the proteomics data under ‘field 30900’ within category 1838 (https://biobank.ndph.ox.ac.uk/showcase/label.cgi?id=1838). Source data are provided with this paper. All other data supporting the findings of this study are available from the corresponding authors upon reasonable request.

## Code availability

Code is available with open access at the following GitHub repository: https://github.com/DanniGadd/Blood_protein_levels_and_incident_disease_UK_Biobank.

## References

[1] Yao C (2018). Genome-wide mapping of plasma protein QTLs identifies putatively causal genes and pathways for cardiovascular disease. Nat. Commun..

[2] Ferkingstad E (2021). Large-scale integration of the plasma proteome with genetics and disease. Nat. Genet..

[3] Pietzner M (2021). Mapping the proteo-genomic convergence of human diseases. Science.

[4] Sun BB (2018). Genomic atlas of the human plasma proteome. Nature.

[5] Gudmundsdottir V (2020). Circulating protein signatures and causal candidates for type 2 diabetes. Diabetes.

[6] Nurmohamed NS (2022). Targeted proteomics improves cardiovascular risk prediction in secondary prevention. Eur. Heart J..

[7] Huth C (2019). Protein markers and risk of type 2 diabetes and prediabetes: a targeted proteomics approach in the KORA F4/FF4 study. Eur. J. Epidemiol..

[8] LaFramboise WA (2012). Serum protein profiles predict coronary artery disease in symptomatic patients referred for coronary angiography. BMC Med..

[9] Georgakis MK, Gill D (2021). Mendelian randomization studies in stroke: exploration of risk factors and drug targets with human genetic data. Stroke.

[10] Ritchie SC (2021). Integrative analysis of the plasma proteome and polygenic risk of cardiometabolic diseases. Nat. Metab..

[11] Sathyan S (2020). Plasma proteomic profile of age, health span, and all-cause mortality in older adults. Aging Cell.

[12] Borrebaeck CAK (2017). Precision diagnostics: moving towards protein biomarker signatures of clinical utility in cancer. Nat. Rev. Cancer.

[13] Hippisley-Cox J, Coupland C, Brindle P (2017). Development and validation of QRISK3 risk prediction algorithms to estimate future risk of cardiovascular disease: prospective cohort study. BMJ.

[14] Williams SA (2019). Plasma protein patterns as comprehensive indicators of health. Nat. Med..

[15] Deelen J (2019). A metabolic profile of all-cause mortality risk identified in an observational study of 44,168 individuals. Nat. Commun..

[16] Ganz P (2016). Development and validation of a protein-based risk score for cardiovascular outcomes among patients with stable coronary heart disease. JAMA.

[17] Wang Z (2019). Metabolomic pattern predicts incident coronary heart disease. Arterioscler. Thromb. Vasc. Biol..

[18] Machado-Fragua MD (2022). Circulating serum metabolites as predictors of dementia: a machine learning approach in a 21-year follow-up of the Whitehall II cohort study. BMC Med..

[19] Eiriksdottir T (2021). Predicting the probability of death using proteomics. Commun. Biol..

[20] Lind L (2021). Large-scale plasma protein profiling of incident myocardial infarction, ischemic stroke, and heart failure. J. Am. Heart Assoc..

[21] Buergel T (2022). Metabolomic profiles predict individual multidisease outcomes. Nat. Med..

[22] Sun BB (2023). Plasma proteomic associations with genetics and health in the UK Biobank. Nature.

[23] Kyu HH (2018). Global, regional, and national disability-adjusted life-years (DALYs) for 359 diseases and injuries and healthy life expectancy (HALE) for 195 countries and territories, 1990–2017: a systematic analysis for the Global Burden of Disease Study 2017. Lancet.

[24] James SL (2018). Global, regional, and national incidence, prevalence, and years lived with disability for 354 diseases and injuries for 195 countries and territories, 1990–2017: a systematic analysis for the Global Burden of Disease Study 2017. Lancet.

[25] Feigin VL (2019). Global, regional, and national burden of neurological disorders, 1990–2016: a systematic analysis for the Global Burden of Disease Study 2016. Lancet Neurol..

[26] Sherwani SI, Khan HA, Ekhzaimy A, Masood A, Sakharkar MK (2016). Significance of HbA1c test in diagnosis and prognosis of diabetic patients. Biomark. Insights.

[27] World Health Organization. Use of glycated haemoglobin (HbA1c) in the diagnosis of diabetes mellitus. Abbreviated report of a WHO consultation. WHO/NMH/CHP/CPM/11.1. apps.who.int/iris/bitstream/handle/10665/70523/WHO_NMH_CHP_CPM_11.1_eng.pdf (2011). 26158184

[28] Li R, Chen Y, Ritchie MD, Moore JH (2020). Electronic health records and polygenic risk scores for predicting disease risk. Nat. Rev. Genet..

[29] Lewis CM, Vassos E (2020). Polygenic risk scores: from research tools to clinical instruments. Genome Med..

[30] Lu AT (2019). DNA methylation GrimAge strongly predicts lifespan and healthspan. Aging (Albany NY).

[31] Bollepalli S, Korhonen T, Kaprio J, Anders S, Ollikainen M (2019). EpiSmokEr: a robust classifier to determine smoking status from DNA methylation data. Epigenomics.

[32] Cheng Y (2023). Development and validation of DNA methylation scores in two European cohorts augment 10-year risk prediction of type 2 diabetes. Nat. Aging.

[33] Barnett JH, Lewis L, Blackwell AD, Taylor M (2014). Early intervention in Alzheimer’s disease: a health economic study of the effects of diagnostic timing. BMC Neurol..

[34] Crous-Bou M, Minguillón C, Gramunt N, Molinuevo JL (2017). Alzheimer’s disease prevention: from risk factors to early intervention. Alzheimers Res. Ther..

[35] Foster LA, Salajegheh MK (2019). Motor neuron disease: pathophysiology, diagnosis, and management. Am. J. Med..

[36] Tanaka T (2020). Plasma proteomic biomarker signature of age predicts health and life span. eLife.

[37] Bao X (2021). Growth differentiation factor-15 is a biomarker for all-cause mortality but less evident for cardiovascular outcomes: a prospective study. Am. Heart J..

[38] Zhang X (2024). Association of a blood-based aging biomarker index with death and chronic disease: Cardiovascular Health Study. J. Gerontol. A Biol. Sci. Med. Sci..

[39] Choy EH (2020). Translating IL-6 biology into effective treatments. Nat. Rev. Rheumatol..

[40] Ridker PM, Rane M (2021). Interleukin-6 signaling and anti-interleukin-6 therapeutics in cardiovascular disease. Circ. Res..

[41] Eugen-Olsen J (2010). Circulating soluble urokinase plasminogen activator receptor predicts cancer, cardiovascular disease, diabetes and mortality in the general population. J. Intern. Med..

[42] Pietzner M (2021). Synergistic insights into human health from aptamer- and antibody-based proteomic profiling. Nat. Commun..

[43] Hastie, T., Tibshirani, R., Narasimhan, B. & Chu, G. impute: imputation for microarray data. R package version 1.60.0. bioconductor.org/packages/impute/ (2022).

[44] Therneau, T. M. A package for survival analysis in R. R package version 3.2-7. CRAN.R-project.org/package=survival (2020).

[45] R Core Team. *R: A Language and Environment for Statistical Computing* (R Foundation for Statistical Computing, 2017).

[46] Chang, W. et al. shiny: web application framework for R. R package version 1.7.3.9002. shiny.posit.co (2024).

[47] Allaire, J. J., Gandrud, C., Russell, K. & Yetman, C. J. networkD3: D3 JavaScript network graphs from R. R package version 0.4. CRAN.R-project.org/package=networkD3 (2017).

[48] Csardi G, Nepusz T (2006). The igraph software package for complex network research. InterJ. Complex Syst..

[49] Simon N, Friedman J, Hastie T, Tibshirani R (2011). Regularization paths for Cox’s proportional hazards model via coordinate descent. J. Stat. Softw..

[50] Greenwell, B., Boehmke, B., Cunningham, J. & GBM Developers. gbm: generalized boosted regression models. R package version 2.1.8.1. CRAN.R-project.org/package=gbm (2022).

[51] Kuhn, M. et al. caret: classification and regression training. R package version 6.0-71. CRAN.R-project.org/package=caret (2016).

[52] Yan, Y. MLmetrics: machine learning evaluation metrics. R package version 1.1.1. CRAN.R-project.org/package=MLmetrics (2016).

[53] Saito T, Rehmsmeier M (2017). Precrec: fast and accurate precision–recall and ROC curve calculations in R. Bioinformatics.

