# Supplementary material for: Blood protein assessment of leading incident diseases and mortality in the UK Biobank
Source: Nat Aging. 2024 Jul 10;4(7):939–48. doi: 10.1038/s43587-024-00655-7 (PMC11257969; doi:10.1038/s43587-024-00655-7)
Supplement: Supplementary file 1 — Supplementary Note. [file 43587_2024_655_MOESM1_ESM.pdf]

nature aging

Supplementary information

https://doi.org/10.1038/s43587-024-00655-7

Blood protein assessment of leading incident diseases and mortality in the UK Biobank

In the format provided by the authors and unedited

Supplementary Information

The Biogen Biobank Team

Denis Baird Research & Development, Biogen Inc. Cambridge, MA US

Danai Chasioti Research & Development, Biogen Inc. Cambridge, MA US

Chia-Yen Chen Research & Development, Biogen Inc. Cambridge, MA US

Susan Eaton Research & Development, Biogen Inc. Cambridge, MA US

Amanda Edwards Research & Development, Biogen Inc. Cambridge, MA US

Kyle L Ferber Research & Development, Biogen Inc. Cambridge, MA US

Jake Gagnon Research & Development, Biogen Inc. Cambridge, MA US

Feng Gao Research & Development, Biogen Inc. Cambridge, MA US

Cynthia Gubbels Research & Development, Biogen Inc. Cambridge, MA US

Yunfeng Huang Research & Development, Biogen Inc. Cambridge, MA US

Megan Jensen Research & Development, Biogen Inc. Cambridge, MA US

Sally John Research & Development, Biogen Inc. Cambridge, MA US

Stephanie Loomis Research & Development, Biogen Inc. Cambridge, MA US

Eric Marshall Research & Development, Biogen Inc. Cambridge, MA US

Helen McLaughlin Research & Development, Biogen Inc. Cambridge, MA US

Adele Mitchell Research & Development, Biogen Inc. Cambridge, MA US

Mehool Patel Research & Development, Biogen Inc. Cambridge, MA US

Heiko Runz Research & Development, Biogen Inc. Cambridge, MA US

Benjamin B Sun Research & Development, Biogen Inc. Cambridge, MA US

Ellen Tsai Research & Development, Biogen Inc. Cambridge, MA US

Romi Admanit Research & Development, Biogen Inc. Cambridge, MA US

Tinchi Lin Research & Development, Biogen Inc. Cambridge, MA US

Christopher D Whelan Research & Development, Biogen Inc. Cambridge, MA US

<u>**Supplementary Text**</u>

Sample selection

A complete summary of the sample selection, processing and quality control details for the UK Biobank PPP proteomics samples is available in Sun *et al*, 2022 [1]. Consortium members chose samples that were enriched for specific diseases of interest. The remainder of the population was randomly sampled through stratified selection against age, sex and recruitment centre. Day of the week of collection, deprivation index and participant ethnicity were confirmed as representative of the wider UK Biobank cohort. Inclusion and eligibility criteria are detailed in: https://biobank.ndph.ox.ac.uk/showcase/showcase/docs/casecontrol_covidimaging.pdf. Of the 1,472 protein levels in the UKB-PPP sample that are used in the present study, 1,463 are unique, due to CXCL8, IL6 and TNF having multiple analyte measurements (annotation information provided in **Supplementary Table 1**). After quality control and removal of outliers, measurements for 52,744 individuals were available. The 1,468 analyte measurements used in this work correspond to 1,459 unique protein levels.

Olink protein technology

Samples were fractioned to 850µl aliquots and stored at -80 °C. Quantification was performed at Olink Analysis Service in Sweden using Olink Proximity Extension Assay technology. Four 384-plex panels were used (cardiometabolic, neurological, inflammatory and oncology) that targeted 1,463 unique proteins. A summary of the protein analytes available with panel information is provided in **Supplementary Table 1**. A full summary of the Olink technology used to generate analyte measurements is detailed in Sun *et al*, 2022 [1]. Panels contained dilution blocks to account for the range of proteins present. Samples were

serially diluted to 1:10, 1:100 and 1:1000 and transferred to the 384-well plates, which had four blocks for each set of 96 samples. Matched antibodies are labelled with complementary oligonucleotides that bind to the target protein in the sample. Hybridization of the probes can thus be recorded through DNA amplification using polymerase chain reaction (PCR 1) to create amplicons for protein assays. Amplicons were combined across each of the four abundance groups, resulting in one well of amplicons per sample. This signal is quantified using next generation sequencing and validated using methods that have been previously reported [2,3].

Details of the inbuilt Olink quality control workflow for Normalized Protein eXpression (NPX) value generation can be accessed through Sun *et al*, 2023 [1]. Briefly, the raw data generated through the Olink quality control workflow (for 54,219 individuals) underwent removal of three control, unprocessed and withdrawn samples. Filtering was then done to remove 1) measurements that had missing NPX values or other QC failures, 2) outlier values that were beyond 5 standard deviations from the mean of the first or second standardized principal component (PCs) and 3) outliers with a median NPX greater than 5 standard deviations from the mean, or an interquaritile range (IQR) greater than 5 standard deviations from the mean IQR. A total of 52,746 individuals with baseline protein measurements were available after the QC processing applied by Sun et al 2023 [1]. Two individuals were excluded due to having withdrawn from the study, leaving 52,744 individuals that were fed into the protein preparation pipeline for the present study, which is summarised in **Extended Data Fig. 2**.

Assessment of technical and genetic effects

To assess the potential impact of protein processing batch (0-7), study centre (1-22) and 20 genetic principal components, protein levels were regressed onto these variables and residuals

were correlated with the original protein levels. Across the 1,468 proteins tested, the lowest Pearson correlation was 0.94, indicating that there was minimal influence of these factors on protein levels. Cox PH models therefore did not incorporate them as covariates. This supports the extensive characterisations from Sun *et al* 2023 previously [1], which suggested that the proteomic data in the UK Biobank PPP sample does not have pronounced plate, batch or study site specific variability.

Summary of incident disease derivation

Cancer diagnoses were sourced from the cancer registry made available by the UK Biobank at (field ID 100092). First occurrence traits made available by the UK Biobank were used to define non-cancer disease diagnoses (field ID 1712). First occurrence traits integrate self-report at baseline with electronic health linkage to ICD9, ICD10 and GP read2/3 codes from healthcare providers across the United Kingdom to identify the earliest date of a given diagnosis for an individual. Self-report data was recorded at the baseline clinic visit through a touchscreen and was then confirmed via verbal interview with a nurse. Any diagnoses included as ICD codes on death registry information (field ID 100093) from the UK Biobank were also integrated. The UK Biobank provides dates of data availability for each data provider at: https://biobank.ndph.ox.ac.uk/ukb/exinfo.cgi?src=Data_providers_and_dates. Censoring dates for cancer outcomes were set to 2016, which is the earliest date of complete data availability across all providers listed in this guidance. Censoring dates were set to October 2021 for non-cancer diseases and November 2021 for death, which were the dates of the data extractions used.

Medication use

Medication self-report at baseline was extracted using fieldID 20003 from the UK Biobank. This covers only a portion of the UKB sample (376,448 individuals), which when subset to

the population of 47,600 with protein measures available in the present study results in 35,073 individuals. To model medication use, 124,198 medication name instances that were recorded in the 35,073 individuals were condensed into unified classes of action, using the anatomical therapeutic chemical (ATC) classification categories. This coding system was previously included in the GWAS of medication classes performed by Wu *et al* [4]. The frequency of these medications grouped into 849 ATC classes in the population of 35,073 individuals is summarised in **Supplementary Table 10**. Blood-pressure lowering medication was defined using the following ATC codes: Antihypertensives (ATC code C02) = 803 individuals, Diuretics (ATC code C03) = 4227 individuals, Beta blockers (ATC code C07) = 3660 individuals, Calcium channel blockers (ATC code C08) = 3674 individuals, Renin-angiotensin system actors (ATC code C09) = 7288 individuals, Statin use (ATC code C10AA) = 8351 individuals. Taken together, 14,074 individuals (of the 35,073) indicated they were taking one or more of the above blood-pressure lowering medications at baseline. This was treated as a binary variable and the comparison with/out adjustment for this variable was performed for ischaemic heart disease Cox PH associations in the subset of 35,073 individuals. Adjustments for age, sex and six lifestyle factors were included in both sets of analyses, with 2,456 cases, 27,468 controls.

MethylPipeR R package information

MethylPipeR is an R package that facilitates systematic and reproducible development of complex trait and incident disease predictors and is available at: https://github.com/marioni-group/MethylPipeR. A user interface for the MethylPipeR package is also available at: https://github.com/marioni-group/MethylPipeR-UI. In previous work, we have applied MethylPipeR to incident type 2 diabetes prediction considering DNA methylation sites as informative features [5]. However, MethylPipeR allows for Cox PH

penalised regression models to be run with for any input features of interest. Input features are provided to the model in the training sample – in this case, the measurements of 1,468 protein analytes available in the UK Biobank PPP consortium sample – and the features that are predictive of the outcome are selected and assigned weighting coefficients. These coefficients can then be applied to the test sample, to project in scores and assess performance.

ProteinScore testing covariate preparation

Three sets of increasingly complex covariates were used to model the difference in AUC resulting from the addition of ProteinScores. In addition to age and sex (that were available for all individuals), an additional set of 24 covariates were considered. These included the six lifestyle covariates modelled in individual Cox PH analyses (BMI, smoking status, alcohol consumption, social deprivation, education status and physical activity). For the extended set, 18 clinically-relevant covariates were selected from the UK Biobank biomarker panel. These have previously been integrated in metabolomics prediction studies of incident disease in the UK Biobank [6] and represent a comprehensive set of measures that are theoretically possible to generate in clinical settings (although generation of all biomarkers is not typically done as part of clinical practice, as disease-specific biomarkers will often be tested in specific circumstances as isolated tests). When the 24 variables were considered across the 47,600 individuals, 4,163 individuals were identified that had >10% missingness and were excluded. In the remaining 43,437 individuals, none of the covariates had >10% missingness and were therefore all retained. Age, sex, six lifestyle covariates and an extended set of 18 covariates were taken forward for ProteinScore testing. Missing covariate information for continuous traits was imputed through knn imputation and these variables were log transformed, whereas categorical and binary variables were imputed through median imputation.

Metabolomics in the UK Biobank

Metabolomics data were available for 12,059 individuals from the population of 47,600 that had proteomic measures available. The NMR assay includes measurement of 168 metabolites and 81 ratios between different combinations of the 168 metabolites; all measures were considered as potentially-informative features for the MetaboScore. Metabolomics data were assessed using the same imputation pipeline as the protein data; nine individuals that had >10% missingness in the 249 metabolomic measures were excluded. In the remaining population of 12,050 individuals, no metabolomic measures had <10% missingness and none were therefore excluded. The dataset was imputed via knn imputation (k=10). Metabolomic measure names were extracted in concordance with those set by the ukbnmr R package (Version 1.5) [7]. Metabolomic data were rank-base inverse normalised and scaled to have a mean of 0 and standard deviation of 1 in the set population subsets used for training and testing (the same approach that was taken to produce the ProteinScore in this population for comparison).

Multimorbidity status individual protein markers

In logistic regression models run between the 1,468 protein analytes and multimorbidity status (a binary trait defined as individuals that had three or more diagnoses of the 23 diseases over the 16-year follow-up period), 720 associations had $P < 3.1 \times 10^{-6}$. All 54 proteins that were associated with eight or more morbidities in the Cox PH associations were present in the multimorbidity status associations. GDF15, TNFRSF10B, WFDC2 and PLAUR had both the largest absolute effect sizes and smallest p-values, which was consistent with their position as top markers of multimorbidity in the individual Cox PH associations.

Cox PH sensitivity by successive yearly intervals

Understanding whether protein-disease associations are stronger in the near-term of case follow-up is of interest when considering the clinical use-case for biomarkers. Modelling near-term versus long-term case follow-up is also important to understand the confidence that can be ascribed to associations failing the Cox PH assumption (Schoenfeld residual test $P < 0.05$). Therefore, each of the 35,232 fully-adjusted Cox PH associations were run over successive yearly intervals of case follow-up. Of the 684 failures in the local (protein) Cox PH assumption observed in the 15-year follow-up analyses, 665 and 410 were observed in the 10-year and 5-year onset analyses. Relatively minor deviations in magnitude of effect size were observed for these associations by year of follow-up.

Cox PH medication sensitivity for ischaemic heart disease

Of 35,073 individuals with medication linkage available, 14,074 reported the use of either statins, antihyperintensives, diuretics, beta blockers, calcium channel blockers or renin-angiotensin system actors at baseline (as defined by ATC criteria). In the subset of 35,073 individuals, 371 of the original 405 associations (adjusting for age, sex and six lifestyle factors) for ischaemic heart disease had $P < 3.1x10^{-6}$**.** With further adjustment for blood-pressure lowering medication use, 336 associations had $P < 3.1x10^{-6}$ (**Supplementary Table 10).**

Supplementary information references

1. Sun, B. B. *et al.* Plasma proteomic associations with genetics and health in the UK Biobank. *Nature* **622**, 329–338 (2023).

2. Wik, L. *et al.* Proximity Extension Assay in Combination with Next-Generation Sequencing for High-throughput Proteome-wide Analysis. *Mol. Cell. Proteomics MCP* **20**, 100168 (2021).
3. Next generation plasma proteome profiling to monitor health and disease | Nature Communications. https://www.nature.com/articles/s41467-021-22767-z.
4. Wu, Y. *et al.* Genome-wide association study of medication-use and associated disease in the UK Biobank. *Nat. Commun.* **10**, 1891 (2019).
5. Cheng, Y. *et al.* Development and validation of DNA methylation scores in two European cohorts augment 10-year risk prediction of type 2 diabetes. *Nat. Aging* **3**, 450–458 (2023).
6. Buergel, T. *et al.* Metabolomic profiles predict individual multidisease outcomes. *Nat. Med.* **28**, 2309–2320 (2022).
7. Ritchie, S.C., Surendran, P., Karthikeyan, S. et al. Quality control and removal of technical variation of NMR metabolic biomarker data in ~120,000 UK Biobank participants | Scientific Data. *Sci. Data* **10**, (2023).
