# Supplementary material for: Blood protein assessment of leading incident diseases and mortality in the UK Biobank
Source: Nat Aging. 2024 Jul 10;4(7):939–48. doi: 10.1038/s43587-024-00655-7 (PMC11257969; doi:10.1038/s43587-024-00655-7)
Supplement: Supplementary file 2 — Reporting Summary [file 43587_2024_655_MOESM2_ESM.pdf]

nature portfolio

Corresponding author(s): Danni Gadd, Dr Ben Sun, Prof Riccardo Marioni

Last updated by author(s): 24/3/24

Reporting Summary

Nature Portfolio wishes to improve the reproducibility of the work that we publish. This form provides structure for consistency and transparency in reporting. For further information on Nature Portfolio policies, see our Editorial Policies and the Editorial Policy Checklist.

Statistics

For all statistical analyses, confirm that the following items are present in the figure legend, table legend, main text, or Methods section.

n/a	Confirmed	
☐	☒	The exact sample size (*n*) for each experimental group/condition, given as a discrete number and unit of measurement
☐	☒	A statement on whether measurements were taken from distinct samples or whether the same sample was measured repeatedly
☐	☒	The statistical test(s) used AND whether they are one- or two-sided<br>*Only common tests should be described solely by name; describe more complex techniques in the Methods section.*
☐	☒	A description of all covariates tested
☐	☒	A description of any assumptions or corrections, such as tests of normality and adjustment for multiple comparisons
☐	☒	A full description of the statistical parameters including central tendency (e.g. means) or other basic estimates (e.g. regression coefficient) AND variation (e.g. standard deviation) or associated estimates of uncertainty (e.g. confidence intervals)
☐	☒	For null hypothesis testing, the test statistic (e.g. *F*, *t*, *r*) with confidence intervals, effect sizes, degrees of freedom and *P* value noted<br>*Give P values as exact values whenever suitable.*
☒	☐	For Bayesian analysis, information on the choice of priors and Markov chain Monte Carlo settings
☒	☐	For hierarchical and complex designs, identification of the appropriate level for tests and full reporting of outcomes
☐	☒	Estimates of effect sizes (e.g. Cohen's *d*, Pearson's *r*), indicating how they were calculated

Our web collection on statistics for biologists contains articles on many of the points above.

Software and code

Policy information about availability of computer code

Data collection	There were no software or code that were used in data collection in the present study. This is because the data resource we used was already available as part of the UK Biobank and had already been collected at the time of analyses starting.
Data analysis	Code is available with open access at the following Github repository: https://github.com/DanniGadd/Blood_protein_levels_and_incident_disease_UK_Biobank. All analyses were performed using this code. The github repository is open access.<br><br>The following software was used:<br>R (Version 4.2.0)<br>impute R package (Version 1.60.0)<br>survival R package (Version 3.4-0)<br>Shiny R package (Version 1.7.3)<br>networkD3 R package (Version 3.0.4)<br>igraph R package (Version 1.3.5)<br>Glmnet R package (Version 4.1-4)<br>gbm R package (Version 2.1.8.1)<br>Caret R package (Version 6.0-94)<br>MLmetrics R package (Version 1.1.1)<br>precrec R package (Version 0.12.9)

For manuscripts utilizing custom algorithms or software that are central to the research but not yet described in published literature, software must be made available to editors and reviewers. We strongly encourage code deposition in a community repository (e.g. GitHub). See the Nature Portfolio guidelines for submitting code & software for further information.

Data

Policy information about availability of data

All manuscripts must include a data availability statement. This statement should provide the following information, where applicable:

- Accession codes, unique identifiers, or web links for publicly available datasets
- A description of any restrictions on data availability
- For clinical datasets or third party data, please ensure that the statement adheres to our policy

Proteomic data were available as part of the UK Biobank Pharma Proteomics Project. Data were collected and housed in the central UK Biobank repository prior to extraction for these analyses. Proteomics data is available in the UK Biobank under Category 1838 at: https://biobank.ndph.ox.ac.uk/ukb/label.cgi?id=1838.
All remaining data used in the present study (i.e. phenotypic, lifestyle, demographic and covariate data used alongside proteomic data) were sourced from the UK Biobank. More information regarding the full measurements available in the UK Biobank can be found at: https://biobank.ndph.ox.ac.uk/showcase/. No further datasets beyond those available through the UK Biobank were used in this study.

All datasets generated in this study are made available in Supplementary Tables.

Human research participants

Policy information about studies involving human research participants and Sex and Gender in Research.

Reporting on sex and gender	We ensure that the use of terms sex and gender is appropriate in the manuscript. We do not use the term gender, as we only utilised biological sex as a covariate in our analyses. We do not have findings that apply to one sex or gender, as sex was modelled as a covariate as questions regarding sex differences in protein signatures of disease were not part of our primary research objectives in this work. All participants of the UK Biobank provided informed consent to share sex status with the cohort resource. Sex is summarised in Supplementary Table 2 (Female = 25,663 individuals [54%], Male = 21,937 individuals [46%]) across the UK Biobank population with protein data that were used in this study.
Population characteristics	Participants included in the analyses (N=47,600) had a mean age of 57.3 years (SD 8.2), with a minimum age of 40.2 and a maximum age of 71. Of these individuals, 4,446 (9%) had died during the 16-year follow-up period after blood samples were taken. Baseline measurements of several covariates were used in fully-adjusted models: BMI (weight in kilograms divided by height in metres squared), alcohol intake frequency (1 = Daily or almost daily, 2 = Three-Four times a week, 3 = Once or twice a week, 4 = One-Three times a month, 5 = Special occasions only, 6 = Never), the Townsend index of deprivation (higher score representing greater levels of deprivation) and smoking status (0 = Never, 1 = Previous, 2 = Current) and education status (1 = college/university educated, 0 = all other education). Of the 47,600 individuals with complete protein data, there were 52, 52, 236, 56 and 59 missing entries for alcohol, smoking, BMI, physical activity and deprivation, respectively. No imputation of missing data was performed for the inclusion of these variables in individual Cox PH analyses. There were an additional 2,556, 188 and 59 individuals that answered 'prefer not to answer' and were excluded from physical activity, smoking and alcohol variables, respectively.
Recruitment	The UKB-PPP sample includes 54,306 UKB participants and 1,474 protein analytes measured across four Olink panels (Cardiometabolic, Inflammation, Neurology and Oncology). A randomised subset of 46,673 individuals were selected from baseline UKB, with 6,385 individuals selected by the UKB-PPP consortium members and 1,268 individuals included that participated in a COVID-19 study. The randomised samples have been shown to be highly representative of the wider UKB population, whereas the consortium-selected individuals were enriched for 122 diseases. All samples analysed in the present study are baseline samples for unique individuals, with 52,744 baseline samples available prior to missingness assessment and imputation steps.
Ethics oversight	All participants provided informed consent. This research has been conducted using the UK Biobank Resource under approved application numbers 65851, 20361, 26041, 44257, 53639, 69804.

Note that full information on the approval of the study protocol must also be provided in the manuscript.

Field-specific reporting

Please select the one below that is the best fit for your research. If you are not sure, read the appropriate sections before making your selection.

☒ Life sciences ☐ Behavioural & social sciences ☐ Ecological, evolutionary & environmental sciences

For a reference copy of the document with all sections, see nature.com/documents/nr-reporting-summary-flat.pdf

Life sciences study design

All studies must disclose on these points even when the disclosure is negative.

Sample size	We used the maximum sample available to us, which was every individual in the UK Biobank that had protein measurements available through the UKB-PPP generation of protein data. The data exclusion section describes the exclusions that were made to the overall maximal

population to result in the 47,600 individuals chosen for this analysis. We chose this group of individuals because they have protein data available to conduct analyses on proteomic signatures associated with incident disease risk. This population was also chosen to facilitate this analysis as the sample has incident disease linkage available. This meant that we had sufficient case counts for the diseases studied to be able to conduct our analyses. There is presently no sample globally that we are aware of that could be used to undertake this study as that of the UKB-PPP sample. Therefore, using the maximal dataset was the logical choice to maximise power to detect statistical associations. The maximal sample of 47,600 individuals was used in individual Cox PH analyses. When selecting train and test sets for ProteinScore development, a case:control ratio of 1:3 was chosen and randomly sampled from the maximal population to avoid the introduction of bias. A ratio of 1:3 cases:controls was chosen to avoid unbalanced case:control data, which can skew test statistics (AUC and PRAUC) and render interpretation uninformative.

Data exclusions

There were 52,744 individuals with baseline measures of proteins available. Of 107,161 related pairs of individuals (calculated through kinship coefficients > 0 across the full UKB cohort), 1,276 pairs were present in these individuals. After exclusion of 104 individuals in multiple related pairs, in addition to one individual randomly selected from each of the remaining pairs, there were 51,562 individuals. A further 3,962 individuals were excluded due to having >10% missing protein measurements. Four proteins that had >10% missing measurements (CTSS.P25774.OID21056.v1 and NPM1.P06748.OID20961.v1 from the neurology panel, PCOLCE.Q15113.OID20384.v1 from the cardiometabolic panel and TACSTD2.P09758.OID21447.v1 from the oncology panel) were then excluded. The remaining 1% of missing protein measurements were imputed by K-nearest neighbour (k=10) imputation using the impute R package (Version 1.60.0) 45. The final dataset consisted of 47,600 individuals and 1,468 protein analytes and was used in the analyses.

Replication

As no cohort has Olink proteomics measured at scale, the Pharma Proteomics Project is unqiue in its magnitude. Therefore, the individual Cox proportional hazards associations could not be replicated in another population, given that sufficient case numbers must occur across the population over successive years of follow-up to run viable models. Therefore, underpowered analyses with low numbers of cases would not suit as a direct replication of these results. A stringent Bonferroni adjustment was used to mitigate against false positives in the absence of replication. Regarding the ProteinScore element of the work, ProteinScore development involved fifty randomised iterations that sampled cases and controls in a 1:3 ratio from the full population. The model that resulted in the median difference in AUC was selected for each trait. The minimum and maximum range in performance across these populations was also reported, such that the consistency of ProteinScores across different train and test populations could be assessed. Although these analyses were not possible to replicate in an alternative cohort, the replication of performance across multiple train/test populations indicates that these scores are unlikely to be driven by underlying population characteristics present in randomly sampled individuals. We welcome replication when Olink datasets that have sufficient electronic health linkage at scale can facilitate this.

Randomization

For individual Cox proportional hazards models, all possible individuals were used and divided into cases and controls based on incident disease status. Therefore, no randomisation was required in this portion of the study.

In the second portion of the study, randimisation was used to undertake training and testing of the ProteinScores. To minimise sample selection biases and assess the consistency of ProteinScore performance across varied populations, fifty seed values were selected at random and used to randomise train/test 50% subsets. Controls were then sampled at random from the populations using a randomised approach in each iteration to give a 1:3 case:control ratio. Each iteration of ProteinScore testing therefore represented a unique combination of individuals across train and test subsets.

Blinding

As this study did not have a clinical trial or intervention format and instead tracked retrospective disease cases through electronic health linkage, no blinding of intervention or patient allocations in groups was needed. Individuals were anonymised as part of the UK Biobank cohort resource, such that they could not be identified during analyses. Proteomic data were processed by individuals that were blinded to the identifiers for individuals and any information on the lifestyle, demographics or health profiles of individuals.

Reporting for specific materials, systems and methods

We require information from authors about some types of materials, experimental systems and methods used in many studies. Here, indicate whether each material, system or method listed is relevant to your study. If you are not sure if a list item applies to your research, read the appropriate section before selecting a response.

Materials & experimental systems

n/a	Involved in the study
☒	☐ Antibodies
☒	☐ Eukaryotic cell lines
☒	☐ Palaeontology and archaeology
☒	☐ Animals and other organisms
☒	☐ Clinical data
☒	☐ Dual use research of concern

Methods

n/a	Involved in the study
☒	☐ ChIP-seq
☒	☐ Flow cytometry
☒	☐ MRI-based neuroimaging
